# Dynamic Characteristics of Composite Sandwich Panel with Triangular Chiral (Tri-Chi) Honeycomb under Random Vibration

**DOI:** 10.3390/ma17163973

**Published:** 2024-08-09

**Authors:** Hui Yuan, Yifeng Zhong, Yuxin Tang, Rong Liu

**Affiliations:** 1 School of Civil Engineering, Chongqing University, Chongqing 400045, China; 202216021133t@stu.cqu.edu.cn (H.Y.); 15823179624@163.com (Y.T.); 202016131332@cqu.edu.cn (R.L.); 2 Key Laboratory of New Technology for Construction of Cities in Mountain Area, Chongqing University, Chongqing 400045, China

**Keywords:** random vibration characteristics, triangular chiral honeycomb, sandwich panel, auxetic effect, variational asymptotic method

## Abstract

A full triangular chiral (Tri-Chi) honeycomb, combining a honeycomb structure with triangular chiral configuration, notably impacts the Poisson’s ratio (PR) and stiffness. To assess the random vibration properties of a composite sandwich panel with a Tri-Chi honeycomb core (CSP-TCH), a two-dimensional equivalent Reissner–Mindlin model (2D-ERM) was created using the variational asymptotic method. The precision of the 2D-ERM in free and random vibration analysis was confirmed through numerical simulations employing 3D finite element analysis, encompassing PSD curves and RMS responses. Furthermore, the effects of selecting the model class were quantified through dynamic numerical examples. Modal analysis revealed that the relative error of the first eight natural frequencies predicted by the 2D-ERM consistently remained below 7%, with the modal cloud demonstrating high reliability. The PSD curves and their RMS values closely aligned with 3D finite element results under various boundary conditions, with a maximum error below 5%. Key factors influencing the vibration characteristics included the ligament–rib angle of the core layer and layup modes of the composite facesheets, while the rib-to-ligament thickness ratio and the aspect ratio exert minimal influence. The impact of the ligament–rib angle on the vibration properties primarily stems from the significant shift in the core layer’s Poisson’s ratio, transitioning from negative to positive. These findings offer a rapid and precise approach for optimizing the vibration design of CSP-TCH.

## 1. Introduction

Materials in nature typically exhibit positive Poisson’s ratios. Nonetheless, there are unique materials called negative Poisson’s ratio (NPR) materials that expand perpendicular to the loading direction, leading to their designation as auxetic materials. Research indicates that auxetic effects are present in various minerals and certain biological tissues [1]. Lakes et al. [2] successfully developed auxetic polymer and metal foams. Polymer foams, known for their lightweight nature and exceptional energy absorption capabilities, have found widespread application in packaging and protective materials. The creation of artificial auxetic materials has seen rapid growth, with a variety of these materials emerging [3,4,5,6,7]. In addition, chiral and anti-chiral structures also exhibit auxetic effects, adding to the diversity of auxetic materials available for research and application [8,9].

**Figure 1:**
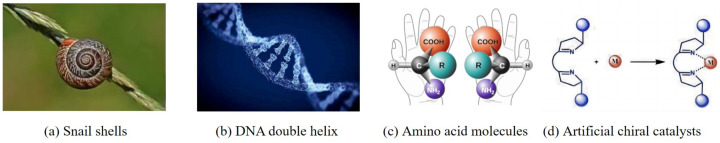
Several chiral structures in nature.

The utilization of cellular material design presents a viable approach for the development of novel lightweight materials with exceptional energy-absorbing capabilities and the creation of metamaterials [10,11]. Cellular metamaterials are characterized by properties not typically observed in natural substances, with their unique properties closely tied to their structure rather than the base material. One intriguing example is the honeycomb metamaterial featuring a negative Poisson’s ratio (NPR), imparting stretchable qualities to the material [12,13,14]. In addition, the chiral metamaterial represents another NPR structure, utilizing a rotational mechanism to achieve its functionality [15,16]. These materials fall under the category of metamaterials possessing periodic arrangements and internal spin overlap characteristics [17,18]. Chirality, a prevalent symmetrical feature in nature, plays a fundamental role in the design of metamaterials. In nature, chirality manifests when the natural structure differs from its mirror image. This phenomenon is illustrated in Figure 1 and underscores the significance of mimicking and harnessing natural asymmetries for the development of innovative materials with unique properties and functionalities.

Cellular structures offer design flexibility by allowing adjustments to the size, shape, and density to meet specific application requirements [19]. Through optimization of these parameters, the performance of the cellular structure can be enhanced. In applications where resistance to vibration and reduction in sound transmission are crucial, the adoption of NPR cellular structures proves beneficial. Among the various NPR cellular structures, chiral honeycombs stand out as effective options [20]. These structures exhibit a unique geometry that enhances their performance, particularly in structural design optimization [21]. Unlike traditional honeycomb structures that undergo concave deformations, the ligaments surrounding the central cylinder in chiral honeycombs rotate, contributing to their exceptional mechanical properties and making them valuable components in a range of applications requiring specialized design features [22].

The unique structural properties exhibited by chiral metamaterials have sparked significant interest in the research community. For instance, Abdeljaber et al. [23] devised an optimization methodology employing genetic algorithms to identify an optimal parameter set. By strategically adjusting the chiral lattice insertions, they effectively mitigated the global vibration levels of a finite-sized beam. Gao et al. [24] delved into the correlation between relative density, topological parameters, and impact energy of chiral structures subjected to impact loads. This study illuminates the impact of various factors on the performance of chiral structures in absorbing impact energy.

Furthermore, Ebrahimi et al. [25] introduced an innovative 3D honeycomb metamaterial that connects planar structures to an anti-chiral topology. This achievement was made possible by incorporating tilt-bearing ligaments to connect circular elements of the anti-chiral topology. Mousanezhad et al. [26] explored the impact of chirality on the in-plane elastic behavior of a 2D honeycomb structure. Their study found that the anti-chiral structure demonstrates both anisotropic characteristics and elongation tendencies as the number of chiral magnetic beads in the cell increases. Despite the extensive research on chiral metamaterials, there is a significant gap in the overall shape alterations due to the predominance of chiral structures produced through ligament design [27]. This limitation impedes the ability to fully explore extensive changes in auxeticity. Therefore, it is imperative to develop design methods for chiral metamaterials that prioritize structural performance and functional characteristics to overcome this limitation and enhance the overall effectiveness of these materials.

**Figure 2:**
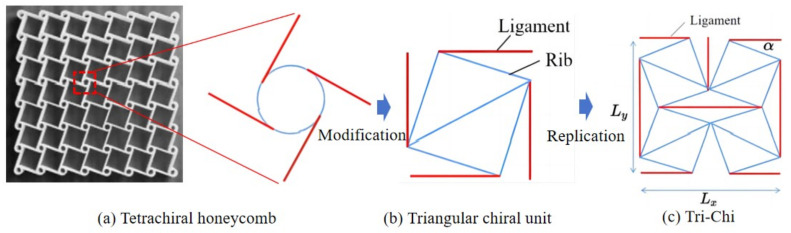
Evolutionary progression of triangular chiral (Tri-Chi) honeycomb structures.

In addition to rotating the square model, Grima et al. [28] revisited the “rotating triangle” mechanism and identified it as a highly effective approach for inducing the auxetic effect. Building upon this concept, Nedoushan et al. [29] developed a triangular chiral structure comprising four cells designed to enhance the stiffness along all primary orientations. This innovative design led to the creation of an extended structure capable of withstanding axial loads, with the initial tetrachiral unit modified by replacing the circular part with a triangular chiral unit, as illustrated in Figure 2b. Subsequently, the cells were duplicated and adjusted in size to create a complete triangular chiral structure known as Tri-Chi, as illustrated in Figure 2c.

It is worth noting that the auxetic properties in 2D in-plane problems and the chiral geometry of the underlying microstructure is the result of the optimization process of minimizing the compliance within the isotropic material design method [30]. The composite sandwich panel with triangular chiral honeycomb core (CSP-TCH) exhibits outstanding deformation resistance due to its unique chiral structure integrated with composite materials. This combination effectively harnesses the benefits of being lightweight, energy absorption, seismic resilience, and sound insulation. Consequently, an investigation into its dynamic characteristics provides vital insights for design considerations. In analyzing the structural dynamic characteristics it is essential to account for random excitations. By examining the random vibration response of the CSP-TCH, one can more accurately evaluate its reliability, allowing for informed design improvements.

Hunady et al. [31] examined the dynamic characteristics of aluminum honeycomb sandwich panels using numerical modal analysis, analyzing the free vibrations of nine panels to assess the impact of geometric parameters (e.g., core thickness and height) on modal characteristics. Hou et al. [32] delved into the energy absorption traits of honeycomb sandwich panels, exploring the influence of material parameters on energy absorption efficacy through experimental and numerical simulations. Ma et al. [33] explored the fatigue performance of composite honeycomb sandwich panels subjected to random vibration loads, investigating the effects of different vibration frequencies and amplitudes on panel fatigue life via experimental and numerical simulations. Presently, the primary research thrust centers on the energy absorption capabilities of honeycomb sandwich structures under dynamic loads [34,35]. Discrepancies in material properties between the facesheet and honeycomb core, coupled with the intricate internal honeycomb core structure prone to pore and defect formation, resulting in material non-uniformity, could impact the reliability and accuracy of vibration characteristics and damping properties [36,37]. Thus, there is a need for appropriate research methodologies and models to tackle these challenges effectively.

This study aims to address the issues by deconstructing the analysis of CSP-TCH into unit-cell level constitutive modeling and a two-dimensional Reissner–Mindlin model using the variational asymptotic method (VAM) with small structural parameters (e.g., thickness to width ratio) [38,39,40]. The ABD matrix derived from the former is applied to the two-dimensional equivalent plate for analyzing dynamic characteristics [41,42]. This approach guarantees precision and efficiency, streamlines model intricacies, lowers computational expenses, and offers significant support for the design of dynamic characteristics and parameter optimization in CSP-TCH.

The paper proceeds as follows: Section 2 presents the theoretical formula and constitutive relationship of 2D-ERM using the VAM. In Section 3, a spectroscopy-based random vibration equation for 2D-ERM is derived. Section 4 validates the effectiveness and precision of 2D-ERM in analyses of free and random vibrations. In addition, Section 5 investigates the impact of critical parameters on the Poisson’s ratio (PR) and random vibration characteristics of CSP-TCH. Section 6 compares the computational efficacy of various models. Finally, Section 7 summarizes the primary conclusions of the study.

## 2. VAM-Based Equivalent Reissner–Mindlin Model for CSP-TCH

**Figure 3:**
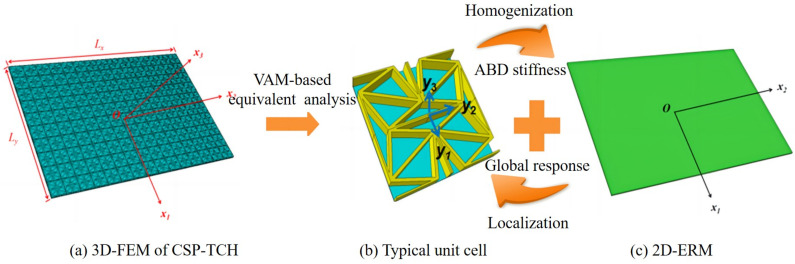
Schematic diagram of equivalent analysis of composite sandwich panels with Tri-Chi honeycomb core (CSP-TCH).

The process of establishing the 2D-ERM of CSP-TCH using the VAM is depicted in Figure 3. The VAM-based 2D-ERM involves the representation of the panel’s displacement field ($u_{i}$) using the displacements (${\bar{u}}_{i}$) in the 2D-ERM and warping functions $w_{i}$, such as

(1) $$\begin{array}{cc} \; & u_{1}\left(x_{1},x_{2},y_{1},y_{2},y_{3},t\right)={\bar{u}}_{1}\left(x_{1},x_{2},t\right)-\zeta y_{3}{\bar{u}}_{3,1}\left(x_{1},x_{2},t\right)+\zeta w_{1}\left(x_{1},x_{2},y_{1},y_{2},y_{3},t\right),\\ \; & u_{2}\left(x_{1},x_{2},y_{1},y_{2},y_{3},t\right)={\bar{u}}_{2}\left(x_{1},x_{2},t\right)-\zeta y_{3}{\bar{u}}_{3,2}\left(x_{1},x_{2},t\right)+\zeta w_{2}\left(x_{1},x_{2},y_{1},y_{2},y_{3},t\right),\\ \; & u_{3}\left(x_{1},x_{2},y_{1},y_{2},y_{3},t\right)={\bar{u}}_{3}\left(x_{1},x_{2},t\right)+\zeta w_{3}\left(x_{1},x_{2},y_{1},y_{2},y_{3},t\right). \end{array}$$

The explicit expressions of ${\bar{u}}_{i}$ can be derived from Equation (1), e.g.,

(2) $${\bar{u}}_{1}=\langle u_{1}\rangle +\zeta \langle y_{3}\rangle {\bar{u}}_{3,1},\;{\bar{u}}_{2}=\langle u_{2}\rangle +\zeta \langle y_{3}\rangle {\bar{u}}_{3,2},\;{\bar{u}}_{3}=\langle u_{3}\rangle ,$$

where 〈·〉 denotes the average volume within the unit cell.

Because the micro-coordinate $y_{i}$ originates from the geometric center of the unit cell, it follows that $\langle y_{3}\rangle =0$ and three constraints are imposed on the warping functions,

(3) $$\langle w_{i}\rangle =0.$$

The concept of rotation tensor decomposition can be used to express the 3D strain components with small local rotation:

(4) $$\varepsilon_{ij}=\frac{1}{2}\left(\frac{\partial u_{i}}{\partial x_{j}}+\frac{\partial u_{j}}{\partial x_{i}}\right).$$

The 3D strain field Γ can be represented in matrix form as

(5) $$\begin{array}{c} \Gamma_{e}={\left[\varepsilon_{11}\;\varepsilon_{22}\;2\varepsilon_{12}\right]}^{T}=\epsilon +\zeta y_{3}\kappa +I_{\alpha }w_{∥,\alpha },\\ 2\Gamma_{s}={\left[2\varepsilon_{13}\;2\varepsilon_{23}\right]}^{T}=w_{∥,3}+e_{\alpha }w_{3,\alpha },\\ \Gamma_{t}=\varepsilon_{33}=w_{3,3}, \end{array}$$

where ${\left(\right)}_{\left|\right|}={\left[{\left(\right)}_{1}\;{\left(\right)}_{2}\right]}^{T}$, $\epsilon ={\left[\begin{array}{ccc} \epsilon_{11} & 2\epsilon_{12} & \epsilon_{22} \end{array}\right]}^{T}$, $\kappa ={\left[\kappa_{11}\;\kappa_{12}+\kappa_{21}\;\kappa_{22}\right]}^{T}$, and

(6) $$I_{1}=\left[\begin{array}{cc} 1 & 0\\ 0 & 1\\ 0 & 0 \end{array}\right],I_{2}=\left[\begin{array}{cc} 0 & 0\\ 1 & 0\\ 0 & 1 \end{array}\right],e_{1}=\left\{\begin{array}{c} 1\\ 0 \end{array}\right\},e_{2}=\left\{\begin{array}{c} 0\\ 1 \end{array}\right\}.$$

**Figure 4:**
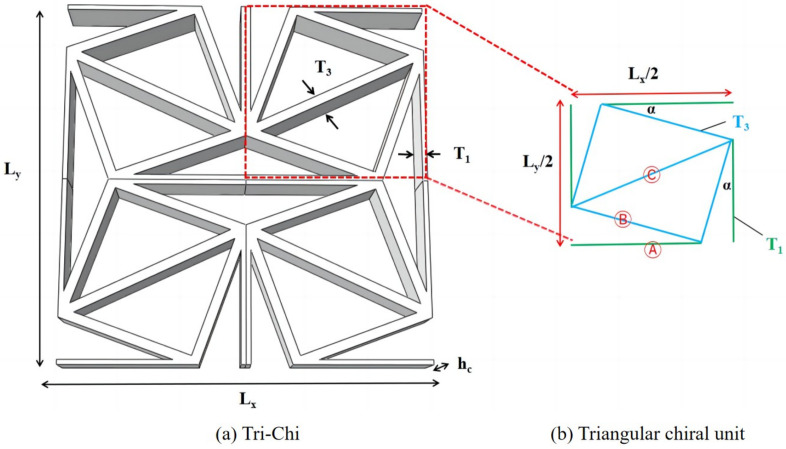
Dimensions of (**a**) core cell of Tri-Chi and (**b**) triangular chiral unit for strain energy integration.

The strain energy can be represented as

(7) $$U=\frac{1}{2}\int_{-a/2}^{a/2}\int_{-b/2}^{b/2}\frac{1}{\Omega }U_{\Omega }dx_{2}dx_{1},$$

where *a* represents the length of the panel and *b* represents the width; $\frac{U_{\Omega }}{\Omega }$ denotes the stain energy density per unit area, and $U_{\Omega }$ can be calculated by adding up the strain energies of individual components,

(8) $$U_{\Omega }=U_{c}+U_{f}=U_{c}+2\times \int_{-\frac{h_{c}}{2}-t_{f}}^{-\frac{h_{c}}{2}}\int_{-\frac{L_{y}}{2}}^{\frac{L_{y}}{2}}\int_{-\frac{L_{x}}{2}}^{\frac{L_{x}}{2}}\Gamma_{f}^{T}D_{f}\Gamma_{f}dy_{1}dy_{2}dy_{3},$$

where $U_{c}$ represents the stain energy of the core cell and $U_{f}$ represents the strain energy of the facesheets,

(9) $$U_{c}=4\times \left(\begin{array}{c} 4\times \int_{-\frac{h_{c}}{2}}^{\frac{h_{c}}{2}}\int_{-\frac{t_{1}}{2}}^{\frac{t_{1}}{2}}\int_{0}^{\frac{L_{x}}{2}-\sqrt{2}r\;\sin\;\alpha }\Gamma_{A}^{T}D_{A}\Gamma_{A}dy_{1}dy_{2}dy_{3}\\ +4\times \int_{-\frac{h_{c}}{2}}^{\frac{h_{c}}{2}}\int_{0}^{-k_{1}y_{1}+\sqrt{2}r\;\sin\;\alpha }\int_{0}^{\frac{L_{x}}{2}-\sqrt{2}r\;\sin\;\alpha }\Gamma_{B}^{T}D_{B}\Gamma_{B}dy_{1}dy_{2}dy_{3}\\ +\int_{-\frac{h_{c}}{2}}^{\frac{h_{c}}{2}}\int_{0}^{k_{2}y_{1}+\sqrt{2}r\;\sin\;\alpha }\int_{0}^{\frac{L_{x}}{2}}\Gamma_{C}^{T}D_{C}\Gamma_{C}dy_{1}dy_{2}dy_{3} \end{array}\right)$$

where subscripts A, B, and C represent the ligament, side rib, and diagonal rib within the core unit, as shown in Figure 4, respectively; *r* denotes the radius of the triangular element; $k_{1}$ and $k_{2}$ refer to the slopes of the B and C ribs, respectively.

Equation (7) can be written as

(10) $$U=\frac{1}{2}\langle \Gamma^{T}D\Gamma \rangle =\frac{1}{2}\langle {\left\{\begin{array}{c} \Gamma_{e}\\ 2\Gamma_{s}\\ \Gamma_{t} \end{array}\right\}}^{T}\left[\begin{array}{ccc} C_{e} & C_{es} & C_{et}\\ C_{es}^{T} & C_{s} & C_{st}\\ C_{et}^{T} & C_{st}^{T} & C_{t} \end{array}\right]\left\{\begin{array}{c} \Gamma_{e}\\ 2\Gamma_{s}\\ \Gamma_{t} \end{array}\right\}\rangle ,$$

where $C_{e},C_{es},C_{et},C_{s},C_{st}$, and $C_{t}$ are the corresponding sub-matrices of the 3D 6×6 material matrix.

### 2.1. First Approximation

The first approximation can be obtained by substituting Equation (5) into Equation (10) and eliminating smaller energy contributions from $w_{i,\alpha }$:

(11) $$2U_{0}=\langle \begin{array}{c} {\left(\epsilon +\zeta y_{3}\kappa \right)}^{T}C_{e}\left(\epsilon +\zeta y_{3}\kappa \right)+2{\left(\epsilon +\zeta y_{3}\kappa \right)}^{T}\left(C_{es}w_{∥,3}+C_{et}w_{3,3}\right)\\ +w_{∥,3}^{T}C_{s}w_{∥,3}+2w_{∥,3}^{T}C_{st}w_{3,3}+w_{3,3}^{T}C_{t}w_{3,3} \end{array}\rangle .$$

Introducing Lagrange multipliers $\lambda_{i}$ allows the associated Euler–Lagrange equations to be derived:

(12) $$\begin{array}{cc} \; & {\left[{\left(\epsilon +\zeta y_{3}\kappa \right)}^{T}C_{es}+w_{∥,3}^{T}C_{s}+w_{∥,3}^{T}C_{st}\right]}_{,3}=\lambda_{∥},\\ \; & {\left[{\left(\epsilon +\zeta y_{3}\kappa \right)}^{T}C_{et}+w_{∥,3}^{T}C_{st}+w_{3,3}C_{t}\right]}_{,3}=\lambda_{3}, \end{array}$$

where $\lambda_{\left|\right|}={\left[\lambda_{1}\;\lambda_{2}\right]}^{T}$.

The free conditions at the top and bottom surfaces can be determined by

(13) $$\begin{array}{cc} \; & {\left[{\left(\epsilon +\zeta y_{3}\kappa \right)}^{T}C_{es}+w_{∥,3}^{T}C_{s}+w_{∥,3}^{T}C_{st}\right]}^{+/-}=0,\\ \; & {\left[{\left(\epsilon +\zeta y_{3}\kappa \right)}^{T}C_{et}+w_{∥,3}^{T}C_{st}+w_{3,3}C_{t}\right]}^{+/-}=0, \end{array}$$

where the superscript “+/−” indicates the quantity being on the top or bottom surface of the panel.

Given these conditions, the solutions for $w_{\left|\right|}$ and $w_{3}$ can be expressed as

(14) $$w_{∥}={\langle -\left(\epsilon +\zeta y_{3}\kappa \right){\bar{C}}_{es}{\left(C_{s}\right)}^{-1}\rangle }^{T},w_{3}=\langle -\left(\epsilon +\zeta y_{3}\kappa \right){\bar{C}}_{et}{\left({\bar{C}}_{t}\right)}^{-1}\rangle ,$$

where

(15) $$\begin{array}{c} {\bar{C}}_{es}=C_{es}-{\bar{C}}_{et}{\left(C_{st}\right)}^{T}{\left({\bar{C}}_{t}\right)}^{-1},\;{\bar{C}}_{et}=C_{et}-C_{es}{\left(C_{s}\right)}^{-1}C_{st},\\ {\bar{C}}_{t}=C_{t}-{\left(C_{st}\right)}^{T}{\left(C_{s}\right)}^{-1}C_{st}. \end{array}$$

The stain energy of the 2D-ERM can be determined by substituting Equation (14) into Equation (11):

(16) $$\begin{array}{c} U_{2D}=\frac{1}{2}\langle {\left(\epsilon +\zeta y_{3}\kappa \right)}^{T}K\left(\epsilon +\zeta y_{3}\kappa \right)\rangle =\frac{1}{2}{\left\{\begin{array}{c} \epsilon \\ \kappa \end{array}\right\}}^{T}\left[\begin{array}{cc} A & B\\ B & D \end{array}\right]\left\{\begin{array}{c} \epsilon \\ \kappa \end{array}\right\} \end{array},$$

with

(17) $$\begin{array}{c} A=\langle K\rangle ,B=\langle \zeta y_{3}K\rangle ,D=\langle \zeta y_{3}^{2}K\rangle ,\\ K=C_{e}-{\bar{C}}_{es}C_{s}^{-1}C_{es}^{T}-{\bar{C}}_{et}C_{et}^{T}/{\bar{C}}_{t}. \end{array}$$

The constitutive relationship for the 2D-ERM can be determined from Equation (16) [43]:

(18) $$\left\{\begin{array}{c} N_{11}\\ N_{22}\\ N_{12}\\ M_{11}\\ M_{22}\\ M_{12} \end{array}\right\}=\left[\begin{array}{cccccc} A_{11} & A_{12} & A_{16} & B_{11} & B_{12} & B_{16}\\ A_{12} & A_{22} & A_{26} & B_{12} & B_{22} & B_{26}\\ A_{16} & A_{26} & A_{66} & B_{16} & B_{26} & B_{66}\\ B_{11} & B_{12} & B_{16} & D_{11} & D_{12} & D_{16}\\ B_{12} & B_{22} & B_{26} & D_{12} & D_{22} & D_{26}\\ B_{16} & B_{26} & B_{66} & D_{16} & D_{26} & D_{66} \end{array}\right]\left\{\begin{array}{c} \epsilon_{11}\\ \epsilon_{22}\\ 2\epsilon_{12}\\ \kappa_{11}\\ \kappa_{22}\\ 2\kappa_{12} \end{array}\right\}.$$

where $A_{ij}$, $B_{ij}$, and $D_{ij}$ are the tensile, coupling, and bending stiffnesses, respectively.

### 2.2. Second Approximation

The first approximation maintains consistency with classical plate theory, enabling the determination of in-plane stresses. To account for out-of-plane stresses, the second approximation becomes essential. This step involves perturbing the warping function as

(19) $$w_{∥}={\bar{v}}_{∥},\;w_{3}={\bar{v}}_{3}+D_{⊥}\chi ,$$

where $\chi ={\left[\begin{array}{cc} \varepsilon & \kappa \end{array}\right]}^{T},D_{⊥}=\left[-\frac{D_{et}^{T}}{D_{t}}-x_{3}\frac{D_{et}^{T}}{D_{t}}\right]$.

Substituting Equation (19) into Equation (5), and then, into Equation (7), the expression for the second approximate energy can be obtained as

(20) $$\begin{array}{cc} 2\Pi_{1}= & \langle {\bar{v}}_{∥,3}^{T}D_{s}{\bar{v}}_{∥,3}+D_{t}{\bar{v}}_{3,3}^{2}+2{\bar{v}}_{∥}^{T}C_{∥,3}\chi_{,\alpha }+2{\bar{v}}_{∥}^{T}D_{s}\partial_{t}D_{⊥}\chi_{,\alpha }-2{\bar{v}}_{∥}^{-T}p_{∥}\rangle \\ \; & -2{\bar{v}}_{∥}^{T}\tau_{∥}-2{\bar{v}}_{∥}^{T}\beta_{∥}. \end{array}$$

The Euler–Lagrange governing equation can be derived as

(21) $${\left(D_{s}{\bar{v}}_{∥,3}+D_{s}\partial_{t}D_{⊥}\chi_{,\alpha }\right)}_{,3}=C_{∥,3}\partial_{t}D_{⊥}\chi_{,\alpha }+g_{,3}+\lambda_{∥},$$

where $C_{∥}=-\partial_{e}^{T}\left[\begin{array}{cc} D_{∥} & x_{3}D_{∥} \end{array}\right],g_{,3}=-p_{∥}$.

Since ${\bar{v}}_{3}$ does not depend on ${\bar{v}}_{∥}$, ${\bar{v}}_{3}$ has a trivial solution. Consequently, the solution for ${\bar{v}}_{∥}$ can be determined by solving Equation (21):

(22) $${\bar{v}}_{∥}=\left({\bar{C}}_{∥}+L_{\alpha }\right)\chi_{,\alpha }+\bar{g},$$

where

(23) $$\begin{array}{c} {\bar{C}}_{∥,3}=D_{s}^{-1}C_{∥},\;\langle {\bar{C}}_{∥}\rangle =0,\;{\bar{g}}_{3}=D_{s}^{-1}\bar{g},\;\langle \bar{g}\rangle =0,\\ L_{\alpha }\chi_{,\alpha }={\bar{C}}_{∥}/h,\;{\bar{C}}_{∥}=C_{∥}+\frac{x_{3}}{h}D_{\alpha }^{\mp }-\frac{1}{2}D_{∥}^{\pm }-D_{s}e_{\alpha }D_{⊥},\\ \bar{g}=g+\frac{x_{3}}{h}g^{\mp }-\frac{1}{2}g^{\pm }, \end{array}$$

and ${\left(\cdot \right)}^{\mp }={\left(\cdot \right)}^{-}-{\left(\cdot \right)}^{+},{\left(\cdot \right)}^{\pm }={\left(\cdot \right)}^{+}+{\left(\cdot \right)}^{-}$.

The second approximate energy can be formulated in the Reissner–Mindlin model as

(24) $$2\Pi_{1}=\chi^{T}A\chi +\chi_{,\alpha }^{T}B_{\alpha \beta }\chi_{,\beta }-2\chi^{T}F,$$

where F is a load-related item:

(25) $$\begin{array}{c} A=\left[\begin{array}{cc} \langle D_{∥}\rangle & \langle x_{3}D_{∥}\rangle \\ \langle x_{3}D_{∥}\rangle & \langle x_{3}^{2}D_{∥}\rangle \end{array}\right],\\ B_{\alpha \beta }=\langle D_{S_{\alpha \beta }}D_{⊥}^{T}D_{⊥}-{\bar{C}}_{\alpha }^{T}D_{s}^{-1}{\bar{C}}_{\beta }\rangle +L_{\alpha }^{T}\left(C_{\beta ,3}\right),\\ F=\langle D_{⊥}^{T}p_{3}\rangle -\langle {\bar{C}}_{∥}^{T}D_{s}^{-1}{\bar{g}}_{,a}\rangle -L_{\alpha }{\left(\langle \bar{p}\rangle +\langle p_{∥}\rangle \right)}_{,\alpha }. \end{array}$$

## 3. Random Vibration Analysis of 2D-ERM

### 3.1. Differential Equation for Random Vibration of 2D-ERM

The dynamic differential equation for the 2D-ERM under lateral random excitation can be expressed as

(26) $$\rho^{*}h{\ddot{\bar{u}}}_{3}\left(x_{1},x_{2},t\right)+c{\dot{\bar{u}}}_{3}\left(x_{1},x_{2},t\right)+D\nabla^{4}{\bar{u}}_{3}\left(x_{1},x_{2},t\right)=p\left(x_{1},x_{2},t\right),$$

where $\nabla^{4}=\frac{\partial^{4}}{\partial x_{1}^{4}}+2\frac{\partial^{4}}{\partial x_{1}^{2}\partial x_{2}^{2}}+\frac{\partial^{4}}{\partial x_{2}^{4}}$ represents the double harmonic operator, $\rho^{*}$ denotes the equivalent density, ${\bar{u}}_{3}\left(x_{1},x_{2},t\right)$ signifies the lateral displacement of the panel, *c* stands for the viscous damping coefficient, *D* represents the equivalent bending stiffness, and $p(x_{1},x_{2},t)$ describes the transverse random excitation, which can be expressed in the form $p\left(x_{1},x_{2},t\right)=\Gamma \left(x_{1},x_{2}\right)X\left(t\right)$, with X(t) denoting a Gaussian stationary random process.

Solving the weak form in Equation (26) typically involves transforming the differential equation into an integral equation through multiplication by a weighting function and applying numerical techniques such as finite element methods or finite difference methods. The resulting system of equations is then solved iteratively to approximate the solution.

### 3.2. Free Vibration Analysis of 2D-ERM

The damping force and the external force on the right-hand side of Equation (26) can be set to zero, leading to the differential equation for the free vibration of 2D-ERM without damping being

(27) $$D\nabla^{4}{\bar{u}}_{3}\left(x_{1},x_{2},t\right)+\rho^{*}h\frac{\partial^{2}{\bar{u}}_{3}\left(x_{1},x_{2},t\right)}{\partial t^{2}}=0$$

The simple harmonic principal vibration of the 2D-ERM is given by ${\bar{u}}_{3}\left(x_{1},x_{2},t\right)=\phi \left(x_{1},x_{2}\right)e^{i\omega t}$, where $\phi (x_{1},x_{2})$ represents the mode shape function; ω is the angular frequency.

Substituting ${\bar{u}}_{3}$ into Equation (27), the mode differential equation can be obtained as

(28) $$D\nabla^{4}\phi -\rho^{*}h\omega^{2}\phi =0$$

For a 2D-ERM with a set of simply supported edges, the mode shape function is

(29) $$\phi \left(x_{1},x_{2}\right)=Y\left(x_{2}\right)\sin\mu x_{1}$$

where μ=mπ/a.

Substituting Equation (29) into Equation (28), a fourth-order ordinary differential equation can be obtained as

(30) $$\frac{d^{4}Y\left(x_{2}\right)}{dx_{2}^{4}}-2\mu^{2}\frac{d^{2}Y\left(x_{2}\right)}{dx_{2}^{2}}+\left(\mu^{4}-\gamma^{4}\right)Y\left(x_{2}\right)=0$$

where $\gamma^{4}=\rho^{*}h\omega^{2}/D$.

The characteristic equation of Equation (30) is written as

(31) $$r^{4}-2\mu^{2}r^{2}+\left(\mu^{4}-\gamma^{4}\right)=0$$

and its four roots are

(32) $$r_{1,2}=\pm \sqrt{\mu^{2}-\gamma^{2}},\;r_{3,4}=\pm \sqrt{\mu^{2}+\gamma^{2}}$$

For the most common case of $\mu^{2}<\gamma^{2}$, the four roots consist of two imaginary roots and two real roots, i.e., $r_{1,2}=\pm i\sqrt{\gamma^{2}-\mu^{2}}=\pm i\alpha ,r_{3,4}=\sqrt{\mu^{2}+\gamma^{2}}=\beta$, then the general solution of Equation (30) is

(33) $$Y\left(x_{2}\right)=A_{1}\cos\alpha +A_{2}\sin\alpha x_{2}+A_{3}\cosh\beta x_{2}+A_{4}\sinh\beta x_{2}$$

where the coefficients of $A_{1}$ to $A_{4}$ can be determined by solving the frequency equation and modal function under the corresponding boundary conditions.

### 3.3. Random Vibration Analysis of 2D-ERM Based on Spectral Method

Using the modal superposition method, the lateral displacement of the 2D-ERM can be represented in modal expansion form as

(34) $${\bar{u}}_{3}\left(x_{1},x_{2},t\right)=\sum_{m=1}^{\infty }\sum_{n=1}^{\infty }\phi_{mn}\left(x_{1},x_{2}\right)\eta_{mn}\left(t\right)$$

where $\phi_{mn}\left(x_{1},x_{2}\right)$ represents the mn-th vibration mode of the 2D-ERM, where *m* and *n* are positive integers, respectively, representing the half-wave number in the 1- and 2-directions, and $\eta_{mn}\left(t\right)$ is the corresponding displacement coordinate of the mn-th vibration mode.

According to modal orthogonality,

(35) $$\int_{0}^{b}\int_{0}^{a}\rho^{*}h\phi_{mn}\left(x_{1},x_{2}\right)\phi_{kl}\left(x_{1},x_{2}\right)dx_{1}dx_{2}=\gamma_{mn}\delta_{mn,kl}$$

(36) $$\int_{0}^{b}\int_{0}^{a}c\phi_{mn}\left(x_{1},x_{2}\right)\phi_{kl}\left(x_{1},x_{2}\right)dx_{1}dx_{2}=c_{mn}\delta_{mn,kl}$$

where $\gamma_{mn}=\int_{0}^{b}\int_{0}^{a}\rho^{*}h\phi_{mn}{\left(x_{1},x_{2}\right)}^{2}dx_{1}dx_{2}$ represents the mass of the mn-th vibration mode; $c_{mn}$ represents the damping ratio of the mn-th vibration mode; $c_{mn}=2\zeta_{mn}\omega_{mn}\gamma_{mn}$, $\omega_{mn}$ denotes the mn-th circular frequency; and $\delta_{mn,kl}$ is the Kronecker delta function.

By multiplying both sides of Equation (26) by the modal shape functions $\phi_{lk}\left(x_{1},x_{2}\right)$, and integrating over the panel surface, one can decouple Equation (26) into a series of single-degree-of-freedom systems:

(37) $${\ddot{\eta }}_{mn}\left(t\right)+2\zeta_{mn}\omega_{mn}{\dot{\eta }}_{mn}\left(t\right)+\omega_{mn}\eta_{mn}\left(t\right)=\frac{1}{\gamma_{mn}}\int_{0}^{b}\int_{0}^{a}p\left(x_{1},x_{2},t\right)\phi_{mn}\left(x_{1},x_{2}\right)dx_{1}dx_{2}$$

The solutions of Equation (37) can be solved using the Duhamel integral:

(38) $$\eta_{mn}\left(t\right)=\frac{1}{\gamma_{mn}}\int_{-\infty }^{\infty }\int_{0}^{b}\int_{0}^{a}h_{mn}\left(t-\tau \right)p\left(x_{1},x_{2},\tau \right)\phi_{mn}\left(x_{1},x_{2}\right)dx_{1}dx_{2}d\tau$$

where $h_{mn}\left(t-\tau \right)$ represents the unit impulse response function.

Substituting Equation (38) into Equation (34), the lateral displacement response can be expressed as

(39) $${\bar{u}}_{3}\left(x_{1},x_{2},t\right)=\sum_{m=1}^{\infty }\sum_{n=1}^{\infty }\phi_{mn}\left(x_{1},x_{2}\right)P_{mn}\int_{-\infty }^{\infty }h_{mn}\left(t-\tau \right)X\left(\tau \right)d\tau$$

where $P_{mn}=\frac{1}{\gamma_{mn}}\int_{0}^{b}\int_{0}^{a}\Gamma \left(x_{1},x_{2}\right)\phi_{mn}\left(x_{1},x_{2}\right)dx_{1}dx_{2}$.

The self-power spectral density of the lateral displacement ${\bar{u}}_{3}\left(x_{1},x_{2},t\right)$ at any point can be expressed as the sum of modal self-correlation terms and modal cross-correlation terms, i.e.,

(40) $$\begin{array}{cc} \; & S_{{\bar{u}}_{3}{\bar{u}}_{3}}\left(x_{1},x_{2},\omega \right)=S_{{\bar{u}}_{3}{\bar{u}}_{3},1}+S_{{\bar{u}}_{3}{\bar{u}}_{3},2}\\ \; & =\sum_{m=1}^{\infty }\sum_{n=1}^{\infty }\phi_{mn}^{2}\left(x_{1},x_{2}\right)P_{mn}^{2}{\left|H_{mn}\left(\omega \right)\right|}^{2}S_{XX}\left(\omega \right)\\ \; & +\sum_{m=1}^{\infty }\sum_{n=1}^{\infty }\sum_{\begin{array}{c} k=1\\ k\neq m \end{array}}^{\infty }\sum_{\begin{array}{c} l=1\\ l\neq n \end{array}}^{\infty }\phi_{mn}\left(x_{1},x_{2}\right)\phi_{kl}\left(x_{1},x_{2}\right)P_{mn}P_{kl}H_{mn}^{*}\left(\omega \right)H_{kl}\left(\omega \right)S_{XX}\left(\omega \right) \end{array}$$

where $H_{mn}\left(\omega \right)={\left(\omega_{mn}^{2}-\omega^{2}+2i\omega \omega_{mn}\zeta_{mn}\right)}^{-1}$ is the frequency response function corresponding to the mn-th frequency; the superscript (*) denotes the complex conjugate.

For the elastic thin panel of the 2D-ERM, the relationship between the stress components and lateral displacements is

(41) $$s_{1}=-\frac{Ex_{3}}{1-v^{2}}\left({\bar{u}}_{3,11}+v{\bar{u}}_{3,22}\right),s_{2}=-\frac{Ex_{3}}{1-v^{2}}\left({\bar{u}}_{3,22}+v{\bar{u}}_{3,11}\right),s_{12}=-\frac{Ex_{3}}{1+v}{\bar{u}}_{3,12}$$

Consequently, the stress self-power spectral density function can be obtained as

(42) $$\begin{array}{cc} S_{s_{1}s_{1}}\left(x_{1},x_{2},\omega \right)= & \frac{E^{2}x_{3}^{2}}{{\left(1-v^{2}\right)}^{2}}\sum_{m=1}^{\infty }\sum_{n=1}^{\infty }\sum_{k=1}^{\infty }\sum_{l=1}^{\infty }\left[\phi_{mn,x_{1}x_{1}}\phi_{kl,x_{1}x_{1}}+v\left(\phi_{mn,x_{1}x_{1}}\phi_{kl,x_{2}x_{2}}+\phi_{mn,x_{2}x_{2}}\phi_{kl,x_{1}x_{1}}\right)\right.\\ \; & \left.+v^{2}\phi_{mn,x_{2}x_{2}}\phi_{kl,x_{2}x_{2}}\right]P_{mn}P_{kl}H_{mn}^{*}\left(\omega \right)H_{kl}\left(\omega \right)S_{XX}\left(\omega \right) \end{array}$$

(43) $$\begin{array}{cc} S_{s_{2}s_{2}}\left(x_{1},x_{2},\omega \right)= & \frac{E^{2}x_{3}^{2}}{{\left(1-v^{2}\right)}^{2}}\sum_{m=1}^{\infty }\sum_{n=1}^{\infty }\sum_{k=1}^{\infty }\sum_{l=1}^{\infty }\left[\phi_{mn,x_{2}x_{2}}\phi_{kl,x_{2}x_{2}}+v\left(\phi_{mn,x_{2}x_{2}}\phi_{kl,x_{1}x_{1}}+\phi_{mn,x_{1}x_{1}}\phi_{kl,x_{2}x_{2}}\right)\right.\\ \; & \left.+v^{2}\phi_{mn,x_{1}x_{1}}\phi_{kl,x}\right]P_{mn}P_{kl}H_{mn}^{*}\left(\omega \right)H_{kl}\left(\omega \right)S_{XX}\left(\omega \right) \end{array}$$

(44) $$S_{s_{12}s_{12}}\left(x_{1},x_{2},\omega \right)=\frac{E^{2}x_{3}^{2}}{{\left(1+v\right)}^{2}}\sum_{m=1}^{\infty }\sum_{n=1}^{\infty }\sum_{k=1}^{\infty }\sum_{l=1}^{\infty }\phi_{mn,x_{1}x_{2}}\phi_{k,x_{1}x_{2}}P_{mn}P_{kl}H_{mn}^{*}\left(\omega \right)H_{kl}\left(\omega \right)S_{XX}\left(\omega \right)$$

where $\phi_{mn,x_{1}x_{1}}$ represents the second-order partial derivative of the mn-th mode with respect to $x_{1}$, and $\phi_{mn,x_{1}x_{2}}$ represents the second-order mixed partial derivative of the mn-th mode with respect to $x_{1}$ and $x_{2}$.

Upon obtaining the self-power spectral densities as presented in Equations (40) and (42) to (44), the mean square value of the arbitrary response $u_{i}\left(x_{1},x_{2},t\right)$ can be obtained by frequency domain integration, i.e.,

(45) $$E\left[u_{i}{\left(x_{1},x_{2},t\right)}^{2}\right]=\int_{0}^{\omega_{c}}S_{u_{i}u_{i}}\left(x_{1},x_{2},\omega \right)d\omega$$

where $\omega_{c}$ is the upper cutoff frequency, and the corresponding response root mean square is

(46) $$\sigma_{i}\left(x_{1},x_{2}\right)=\sqrt{\int_{0}^{\omega_{c}}S_{u_{i}u_{i}}\left(x_{1},x_{2},\omega \right)d\omega }$$

## 4. Model Validation

This section presents a comparative analysis between the outcomes of a 3D finite element analysis (3D-FEA) and the VAM-based 2D equivalent Reissner–Mindlin model (2D-ERM) to assess its accuracy in analyzing the dynamic characteristics of CSP-TCH. The effects of selecting the model class are quantified through dynamic numerical examples [44], wherein the relative error signifies the variance between the 2D-ERM and 3D-FEA outcomes. The dynamic analyses of both models are implemented using the linear perturbation procedure (frequency and random response) in the Abaqus finite element software. The dimensions of the benchmark sandwich panel are *a* = 450 mm and *b* = 300 mm, and $T_{1}=4\;\mathrm{mm}$, $T_{3}=8\;\mathrm{mm}$, $\alpha =20^{\circ }$, $L_{x}=L_{y}=15\;\mathrm{mm}$, as shown in Figure 4b. The facesheet height is $h_{c}$ = 1 mm, with a height ratio of 10:1 between the core layer and facesheet ($h_{c}$:$h_{f}$).

The core layer is constructed from aluminum, an isotropic material with material properties of $\rho =2.7\;g/{\mathrm{cm}}^{3}$, $E_{AL}=2.7\;\mathrm{GPa}$, and $\nu_{AL}=0.3$. On the other hand, the facesheet is composed of CFRP (T800), arranged in layup mode of ${\left[45/-45/0/90\right]}_{s}$. The homogenized material parameters of the CFRP facesheet are presented in Table 1 for reference.

### 4.1. Free Vibration Verification

**Figure 5:**
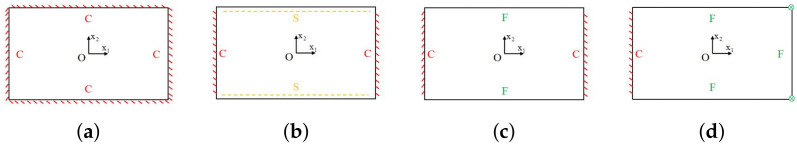
Boundary conditions for dynamic analysis of CSP-TCH. (**a**) Case 1: CCCC. (**b**) Case 2: SSCC. (**c**) Case 3: FFCC. (**d**) Case 4: FFCF.

To investigate the dynamic performance of the CSP-TCH and access the accuracy of 2D-ERM, different cases were selected for numerical simulation comparison, as shown in Figure 5. In this context, “F”, “S”, and “C” denote freely, simply, and clamp-supported edges, respectively. The naming convention reflects the combination of opposite sides, for instance, “FFCC” implies that the right and left sides are fixed while the upper and lower sides are free sides.

Table 2 compares the first eight natural frequencies and free vibration modes of CSP-TCH obtained from the 3D-FEA and 2D-ERM under CCCC boundary conditions (BCs). It is clear that as the modal order increases, the vibration modes become more complex. Notably, except for the third, fifth, eighth, and tenth modes, where lateral deflections occur, the remaining modes predominantly align along the 2-direction as the lateral deflections are zero on all four sides. This inclination is due to the larger size of the 2-direction compared to the 1-direction, emphasizing the importance in engineering design of loading towards the smaller side to mitigate strong resonance effects. The modal comparison highlights a close correspondence between the vibration modes of the 3D-FEA and 2D-ERM, signifying that the 2D-ERM not only accurately predicts natural frequencies but also effectively captures vibration modes. This agreement lays a solid groundwork for subsequent random vibration analysis based on modal superposition.

Table 3 presents the first eight frequencies predicted by the 2D-ERM and 3D-FEA across the other three BCs. The comparison reveals a high degree of consistency in the first eight frequencies between the 2D-ERM and 3D-FEA. Owing to spatial constraints, the detailed listing of high-order modes under the SSCC, FFCC, and FFCF BCs is omitted. Nonetheless, it is anticipated that the visualization of high-order free vibration modes under the three aforementioned BCs mirrors that under CCCC BCs in Table 2. Furthermore, a marginal disparity in natural frequencies between the 3D-FEA and 2D-ERM is observed, with a notable alignment in mode shapes.

### 4.2. Random Vibration Verification

**Figure 6:**
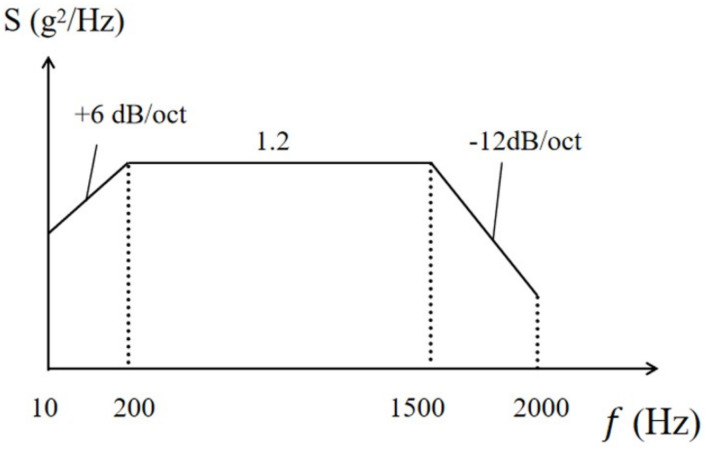
Power spectral density curve of random excitation.

Introducing random loads facilitates a comprehensive analysis of CSP-TCH’s random vibration behavior, further validating the efficacy of the equivalent model in assessing its dynamic characteristics [45]. In the random vibration analysis, the random excitation detailed in Table 4 is utilized, and its PSD curve is illustrated in Figure 6. The analysis incorporates direct modal damping with a damping ratio set at 0.05. Focusing on the first eight natural frequencies and associated vibration modes of the panel, a comprehensive random vibration response analysis is conducted. This analysis encompasses parameters such as the PSD curve, effective mass fraction, RMS value, etc. The study considers four boundary conditions without out-of-plane loading: CCCC (0–2000 Hz), SSCC (0–1800 Hz), FFCC (0–1100 Hz), and SCCS (0–700 Hz).

#### 4.2.1. Power Spectral Density Response

**Figure 7:**
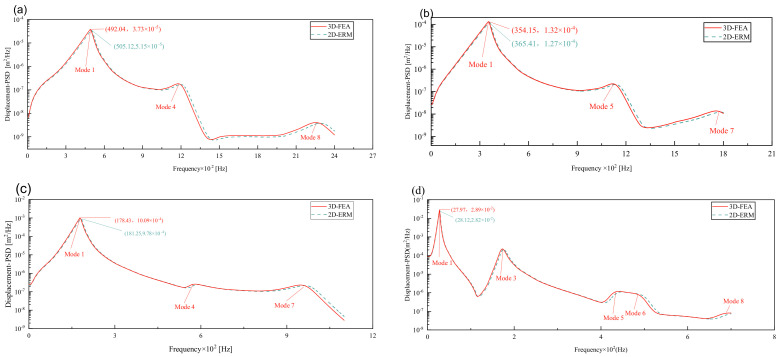
Comparison of displacement PSD curves predicted by 2D-ERM and 3D-FEA under (**a**) case 1: CCCC, (**b**) case 2: SSCC, (**c**) case 3: FFCC, and (**d**) case 4: FFCF.

Figure 7 compares the displacement PSD curves at the center point of the panel under four BCs, as predicted by the 3D-FEA and 2D-ERM. Notably, the displacement PSD curves exhibit good alignment across all BCs, with the error in peak displacement PSD under FFCF BCs at 2.42%, notably lower than the 4.29% error observed under CCCC BCs. The peak displacement PSD value reaches 3.73 × 10^−5^ m under CCCC BCs, while the maximum peak value occurs under FFCF BCs. The trend indicates that heightened boundary constraints lead to reduced displacement responses but with larger peak errors. Consequently, reinforcing boundary constraints in engineering applications can effectively mitigate issues associated with substantial resonance-induced displacements.

**Figure 8:**
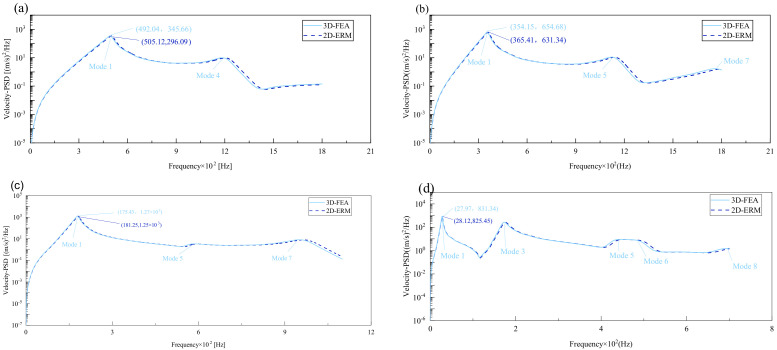
Comparison of displacement PSD curves predicted by 2D-ERM and 3D-FEA under (**a**) case 1: CCCC, (**b**) case 2: SSCC, (**c**) case 3: FFCC, and (**d**) case 4: FFCF.

Figure 8 compares the velocity PSD curves across the four cases, revealing that the excitation frequency associated with the maximum peak closely aligns with the fundamental frequency of free vibration obtained through modal analysis, albeit it is slightly lower. This discrepancy is attributed to Rayleigh damping, which causes the resonance frequency to be marginally lower than the panel’s natural frequency. Notably, the number of peaks differs across BCs, with two peaks under CCCC BCs, three under SSCC and FFCC BCs, and five under FFCF BCs. This pattern corresponds well with the effective mass fractions derived from the 3D-FEA and 2D-ERM. In addition, within the same case, modes possessing higher effective mass fractions exhibit greater peak values in velocity and displacement PSD curves. This observation underscores the significance of the effective mass fraction in elucidating mode behaviors.

**Figure 9:**
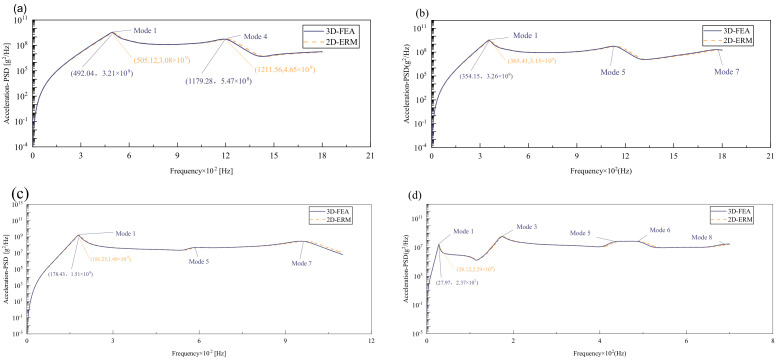
Comparison of acceleration PSD curves predicted by 2D-ERM and 3D-FEA under (**a**) case 1: CCCC, (**b**) case 2: SSCC, (**c**) case 3: FFCC, and (**d**) case 4: FFCF.

Figure 9 compares the acceleration PSD curves of CSP-TCH under four cases as predicted by the 2D-ERM and 3D-FEA. The number and distribution of peak values in the acceleration PSD curve aligns with those of effective mass fractions under varying conditions. For instance, the highest peak may lie in the first mode of the acceleration PSD under CCCC BCs, the fourth mode remains substantial, indicating a heightened influence of other peaks on the acceleration PSD curve, contrasting the patterns observed in displacement- and velocity PSD curves. Furthermore, the predominant peak occurs at the fourth mode under FFCF BCs, surpassing the magnitude of the first peak significantly. This observation suggests that the acceleration PSD curve diverges from the trend where larger effective mass fractions correspond to larger responses, characteristic of displacement- and velocity PSD curves. Despite distinct acceleration PSD behavior, the predictions from the 2D-ERM align closely with those from the 3D-FEA, meeting the requirements of engineering precision.

Table 5 compares PSD errors under different cases. It can be seen that the maximum error occurs in the peak displacement PSD under CCCC BCs, which is 4.29%. The smallest error occurs at the peak of the velocity PSD under FFCF BCs, which is only 0.71%. It is observed that under different BCs, with the weakening of boundary constraints, the error also decreases, which accords with the trend that strong boundary constraints correspond to larger overall errors. In general, all the errors are within 5%, to meet the requirements of engineering accuracy. This shows that the 2D-ERM performs well in predicting structural dynamic response, and its prediction results are within a reasonable error range with those of 3D-FEA even under complex boundary conditions, and its reliability is verified.

#### 4.2.2. Effective Mass Fraction

Effective mass fraction serves as a critical metric for exploring random vibration responses. It aids in the analysis of plate vibration characteristics and structural reliability assessment. Principal modes are characterized by non-zero effective mass fractions, with higher fractions indicating modes that are more easily excited and displaying larger oscillation peaks. Table 6 compares the effective mass fractions predicted by the 3D-FEA and 2D-ERM under four BCs. The total mass of the 3D-FEA is 3.1900188 × 10^−3^, while for the 2D-ERM it is 3.1900561 × 10^−3^. Due to the presence of pores in the 3D-FEA, its total mass is slightly lower than that of the 2D-ERM. The distribution of effective mass fraction aligns closely with the amplitude variation of random vibration: notably, the maximum amplitude occurs at the first order for all four BCs. This phenomenon arises from the fact that the first effective mass fraction is the largest, contributing significantly to random load response. Moreover, the fundamental frequency exerts the most substantial impact on the effective mass fractions. Therefore, when analyzing the random vibration response of the panel, it is crucial to focus on the fundamental frequency and conduct thorough analysis and evaluation accordingly.

#### 4.2.3. Root Mean Square Response

On the basis of verifying the PSD response of CSP-TCH, this section further examines the RMS response, which holds greater significance for its random vibration characteristics. Table 7 compares the displacement, velocity and acceleration RMS clouds at the center point of the panel under four BCs. The analysis reveals a striking similarity in the RMS clouds generated by both models, with a negligible error margin of only 2.45%, meeting established engineering criteria. Inspection of the RMS clouds indicates that symmetric boundary conditions result in symmetric RMS clouds, while variations in boundary conditions cause shifts in the peak position of the RMS cloud. This observation signifies the potential value of analyzing RMS clouds to assess vibration characteristics and energy distribution of the panel, thereby offering insights for structural optimization in the design processes. Notably, the RMS cloud of random vibration under the four BCs aligns with the first free vibration mode. This mode holds exceptional significance, exerting a substantial influence on the overall vibration response due to its distinctive vibrational characteristics.

**Figure 10:**
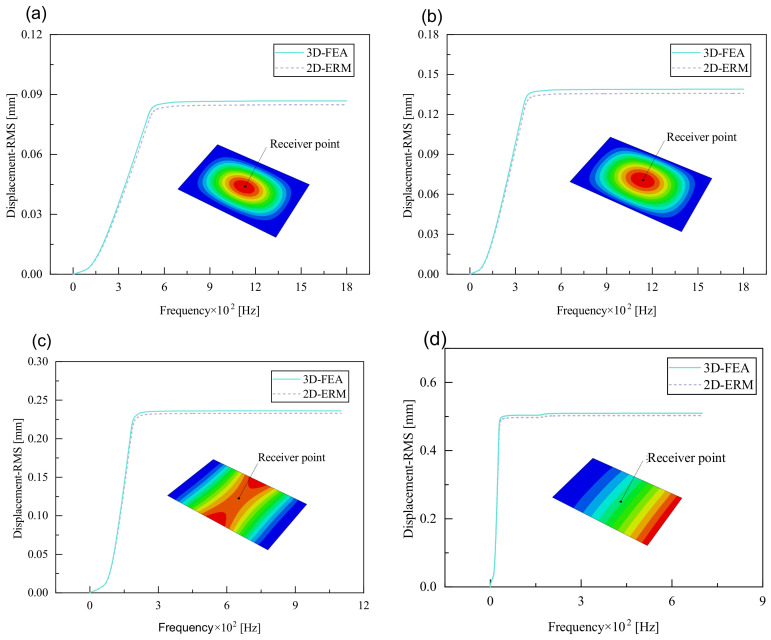
Comparison of displacement RMS curves at the receiver point predicted by 2D-ERM and 3D-FEA under (**a**) case 1: CCCC, (**b**) case 2: SSCC, (**c**) case 3: FFCC, and (**d**) case 4: FFCF.

**Figure 11:**
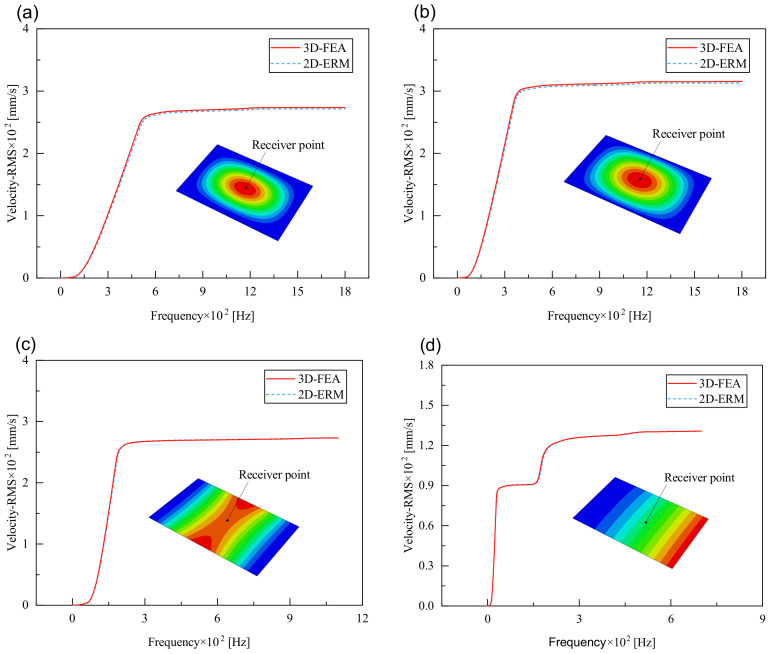
Comparison of velocity RMS curves at the receiver point predicted by 2D-ERM and 3D-FEA under (**a**) case 1: CCCC, (**b**) case 2: SSCC, (**c**) case 3: FFCC, and (**d**) case 4: FFCF.

**Figure 12:**
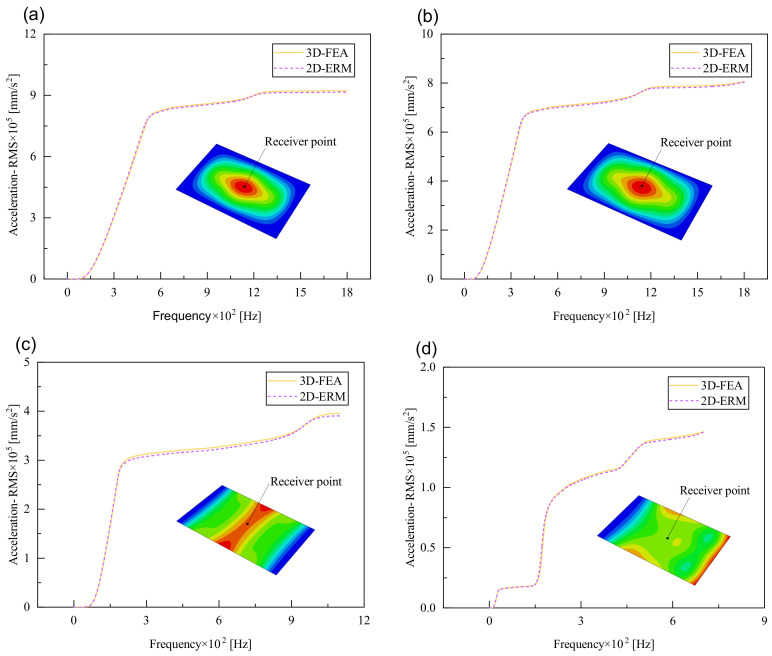
Comparison of acceleration RMS curves at the receiver point predicted by 2D-ERM and 3D-FEA under (**a**) case 1: CCCC, (**b**) case 2: SSCC, (**c**) case 3: FFCC, and (**d**) case 4: FFCF.

Figure 10, Figure 11 and Figure 12 compare the RMS curves at the pickup point located at the center of the panel, as predicted by the 2D-ERM and 3D-FEA under different BCs. The trends in change projected by the 2D-ERM align closely with the outcomes from the 3D-FEA, demonstrating minimal discrepancies with a maximum error of 2.45%. Furthermore, the results indicate that the displacement and velocity RMS values exhibit a linear and pronounced increase with increasing frequency, followed by a gradual deceleration after reaching peak values. Notably, the frequency corresponding to the first turning point of each curve is close to the fundamental frequency under the corresponding BCs. The velocity RMS curve under FFCF BCs, as illustrated in Figure 11d, displays a unique behavior where it does not immediately flatten out after reaching the first turning point. Instead, it continues to rise to a new peak before gradually stabilizing. This distinctive phenomenon can be attributed to the effective mass fraction associated with FFCF BCs. Analysis of the data presented in Table 6 reveals that the third mode holds a relatively substantial share in the effective mass fraction under FFCF BCs, accounting for close to 20%. The heightened effective mass fraction of the third mode signifies its pronounced influence on the structural vibration. Consequently, the velocity RMS curve demonstrates a distinct change pattern, setting it apart from the curves observed under other three BCs. This observation underscores the impact of different vibration modes on the dynamic characteristics of the panel, emphasizing the significant effect that the effective mass fraction exerts on the vibration response. Figure 12 shows the acceleration RMS curves under four BCs, which do not immediately flatten out upon reaching the first turning point. This behavior is linked to the heightened susceptibility of the acceleration PSD to higher vibration modes, as previously discussed. However, as the boundary constraints intensify, this susceptibility diminishes, indicating a trend where stronger constraints lead to a more stabilized acceleration RMS curve after the first turning point. For instance, the last turning point of the acceleration RMS curve corresponds to a higher frequency value within its frequency range under SSCC BCs. Conversely, lower frequency values correspond to CCCC BCs. This pattern illustrates that stronger boundary constraints result in a reduced impact of higher-order modes on the acceleration RMS values. Consequently, accounting for the influence of boundary conditions in engineering applications stands as a critical factor in guaranteeing the performance of the CSP-TCH.

## 5. Influence of Critical Parameters on Poisson’s Ratio and Random Dynamic Characteristics

Based on the validated equivalent model, this section examines the impact of key parameters (e.g., ligament–rib angle, thickness ratio $T_{1}/T_{3}$, width–height ratio $L_{x}/h_{c}$, and layup mode) on the PR and random vibration characteristics of the sandwich panel, focusing primarily on the displacement PSD and displacement RMS under CCCC BCs.

### 5.1. Ligament–Rib Angle

**Figure 13:**
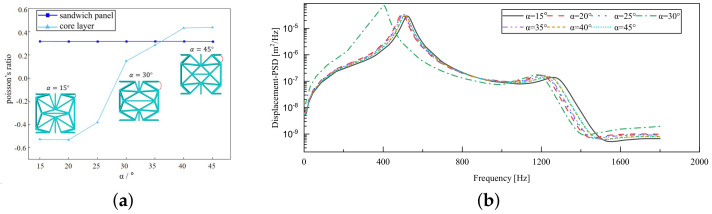
Influence of ligament–rib angle on the Poisson’s ratio (PR) and displacement PSD of CSP-TCH. (**a**) Poisson’s ratio. (**b**) Displacement PSD curve.

Figure 13a illustrates that with the ligament–rib angle gradually increasing from 15° to 45°, the PR of the sandwich panel fluctuates around 0.317, suggesting that the change in the ligament–rib angle has minimal impact on it. The PR of the core layer generally increases, shifting from a negative to a positive value. The discrepancy in PR alterations between the core layer and the sandwich panel is due to the fact that the high stiffness of the facesheet limits the lateral deformation of the core layer. This disparity highlights the intricate interplay among the different components of the sandwich panel during the fluctuation process.

Figure 13b illustrates the displacement PSD curves corresponding to various ligament–rib angles, reflecting a consistent peak at approximately 510 Hz, in alignment with the fundamental frequency. The structural stiffness proportionally rises with an increase in the ligament–rib angle, leading to a gradual decrease in the displacement PSD values. Significantly, a discernible deviation emerges in the displacement PSD curve at $\alpha =30^{\circ }$, with a premature peak at 400 Hz that surpasses values at other angles. This discrepancy can be attributed to a shift in PR from negative to positive at the $30^{\circ }$ angle, as illustrated in Figure 13a. This shift likely triggers local resonance within the panel, impacting the transmission and diffusion of vibrational energy, and subsequently, amplifying the vibration displacement of the panel.

### 5.2. Thickness Ratio $T_{1}/T_{3}$

**Figure 14:**
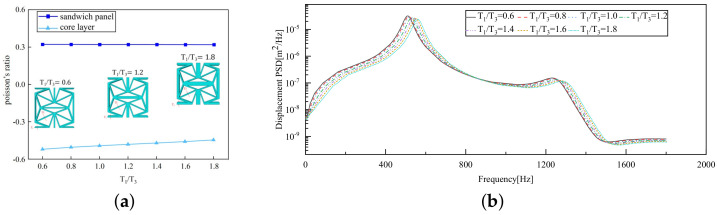
Influence of thickness ratio on the Poisson’s ratio (PR) and displacement PSD of CSP-TCH. (**a**) Poisson’s ratio. (**b**) Displacement PSD curve.

Figure 14a depicts that the PR of the core layer rises steadily as the thickness ratio escalates from 0.6 to 1.8, shifting from −0.521 to −0.446. This suggests that while the core layer maintains the NPR effect, an increase in the ratio diminishes the likelihood of the core layer flipping along the cross-section, thereby lowering the NPR value. The high stiffness of the facesheet ensures that the PR of the sandwich panel remains relatively constant at around 0.33, regardless of any changes in the ligament–rib angle.

Figure 14b illustrates that the displacement PSD curve resulting from variations in the $T_{1}/T_{3}$ ratio exhibits a consistent pattern. Unlike the impact of the ligament–rib angle, alterations in this ratio do not lead to sudden changes. The curve consistently peaks around 510 Hz, consistent with the fundamental frequency. The peak displacement PSD value occurs at $T_{1}/T_{3}=0.6$, with the minimum observed at $T_{1}/T_{3}=1.8$. As the $T_{1}/T_{3}$ ratio increases, there is a gradual decrease in the displacement PSD value. This trend supports the notion that a higher $T_{1}/T_{3}$ ratio corresponds to greater structural stiffness and reduced vibrational response. In practical engineering applications, tailoring the $T_{1}/T_{3}$ ratio can lead to lower displacement PSD values, thus mitigating significant resonance displacement in the panel and enhancing its overall stability.

### 5.3. Width–Height Ratio $L_{x}/h_{c}$

**Figure 15:**
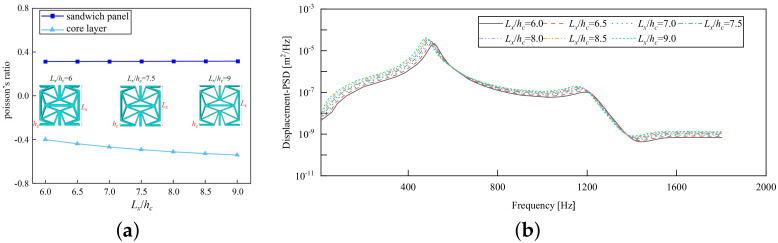
Influence of width–thickness ratio on the Poisson’s ratio (PR) and displacement PSD of CSP-TCH. (**a**) Poisson’s ratio. (**b**) Displacement PSD curve.

Figure 15a illustrates that the PR of the core layer decreases gradually as the $L_{x}/h_{c}$ ratio increases from 6.0 to 9.0, shifting from −0.41 to −0.54. This phenomenon occurs because when the width of the unit cell surpasses the thickness of the core layer, expanding the width while maintaining the core layer thickness leads to an increase in transverse deformation within the core layer. Consequently, this increment in transverse deformation results in the increasing NPR of the core layer. The PR of the sandwich panel stays constant irrespective of changes in the width–height ratio.

Figure 15b illustrates the displacement PSD curves corresponding to various $L_{x}/h_{c}$ ratios, demonstrating a consistent pattern that peaks around 510 Hz, reflecting the fundamental frequency of the sandwich panel. Notably, the displacement PSD value reaches its minimum at $L_{x}/h_{c}=0.6$; whereas it reaches its peak at $L_{x}/h_{c}=1.8$. The displacement PSD value consistently rises with an increase in the $L_{x}/h_{c}$ ratio, in line with the trend of decreasing structural stiffness as the $L_{x}/h_{c}$ ratio increases. In engineering applications, reducing the $L_{x}/h_{c}$ ratio can enable the attainment of a smaller displacement PSD value, thereby preventing significant resonance displacement and enhancing the overall stability of the panel.

### 5.4. Layup Mode

**Figure 16:**
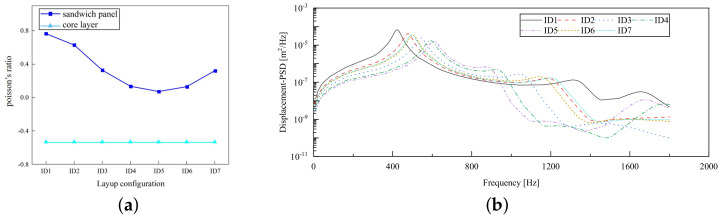
Influence of layup mode on the Poisson’s ratio (PR) and displacement PSD of CSP-TCH. (**a**) Poisson’s ratio. (**b**) Displacement PSD curve.

The stiffness of the composite sandwich panel can be modified by altering the facesheet’s layup modes, consequently influencing its PR. This adjustment occurs because the layup arrangement impacts the fiber orientation within the facesheet, subsequently influencing the overall stiffness properties of the composite sandwich panel. To study this effect, seven layup modes were chosen for investigation: ID1: ${\left[\pm 30\right]}_{2s}$; ID2: ${\left[\pm 45\right]}_{2s}$; ID3: ${\left[\pm 60\right]}_{2s}$; ID4: ${\left[\pm 75\right]}_{2s}$; ID5: ${\left[90\right]}_{2s}$; ID6: ${\left[0/90\right]}_{2s}$; ID7: ${\left[45/-45/0/90\right]}_{s}$. Figure 16a shows that the PR of the sandwich panel decreases progressively with an increase in the fiber layup angle from ID1 to ID5. This change arises because the stiffness of the laminated facesheet is primarily dictated by the orientation of the internal fibers, leading to a reduction in the PR when multiple angles are implemented. Conversely, the PR of the core layer remains relatively constant regardless of changes in the layup angle, as the core layer’s geometry remains consistent.

Figure 16b depicts the impact of fiber layup mode on the displacement PSD of CSP-TCH under CCCC BCs. Notably, ID1 exhibits the most significant displacement PSD peak, whereas ID5 exhibits the smallest peak. Specifically, the curve trajectories of ID2, ID6, and ID7 closely align. This coherence arises from the similarities in the ABD stiffness matrix of the panel across these three layup modes. An important observation is that for ID7 the peak displacement PSD value of the first mode falls in the mid-range, with the corresponding frequency also being intermediary. In contrast, ID4 exhibits a smaller peak displacement PSD but necessitates the highest frequency. Consequently, opting for the ID7 layup proves advantageous in mitigating low-frequency resonance occurrences by yielding a relatively moderate peak displacement PSD. This highlights the commendable structural performance associated with this specific layup mode.

### 5.5. Influence of Critical Parameters on Displacement RMS

**Figure 17:**
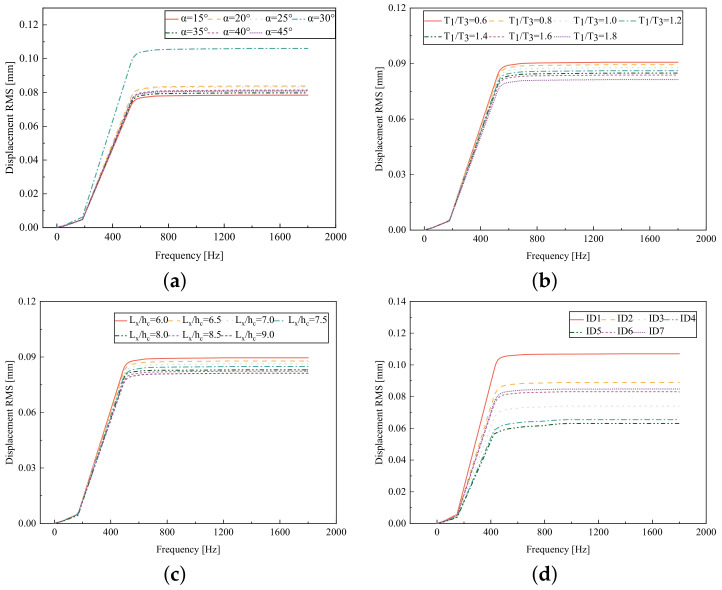
Comparison of displacement RMS curves predicted by 2D-ERM and 3D-FEA under four critical parameters. (**a**) α. (**b**) $T_{1}/T_{3}$. (**c**) $L_{x}/h_{c}$. (**d**) Layup modes.

Figure 17 illustrates that the displacement RMS of CSP-TCH remains consistent despite variations in critical parameters. Specifically, the displacement RMS increases with the increase in the width–thickness ratio ($L_{x}/h_{c}$) and ligament–rib angle, as well as decreases in the thickness ratio ($T_{1}/T_{3}$). It is noteworthy that $L_{x}/h_{c}$ and $T_{1}/T_{3}$ have minimal influence on the displacement RMS compared to the ligament–rib angle. Crucially, a transition in the ligament–rib angle from 25° to 30° results in a sudden spike in the displacement RMS of CSP-TCH. This abrupt change is attributed to the shift in the PR of the core layer from negative to positive, triggering a localized resonance phenomenon during random vibration and significantly magnifying the displacement alteration.

Figure 17d offers a visual representation of the displacement RMS of CSP-TCH across various layup modes. It is evident that ID1 exhibits the highest displacement RMS value, ID5 displays the lowest, and ID7 falls in between, highlighting the significant impact of different layup modes on the sandwich panel’s vibration characteristics. This trend is attributed to the alteration in panel stiffness resulting from the increasing ply angle, which subsequently influences the panel’s natural frequency and vibration mode.

### 5.6. Summary

For a detailed analysis of the diverse impact of each parameter, Table 8 compares the change rates of equivalent density ($\rho^{*}$), PR of the core layer ($\nu_{1}$) and sandwich panel ($\nu_{2}$), first-order natural frequency (ω), first-order displacement RMS ($RU_{3}$), and displacement PSD ($U_{3}$) under the impact of individual parameters.

Table 8 highlights that critical parameters impacting the vibration characteristics include the ligament–rib angle and facesheet layup mode, whereas the thickness ratio and the width–height ratio have relatively minimal effects. It is crucial to recognize that different parameters have distinct impacts on the structural vibration characteristics. For instance, the thickness ratio and width–height ratio primarily affect vibration characteristics through variations in the panel’s equivalent density. Conversely, the influence of the ligament–rib angle on vibration characteristics stems from significant changes in the PR of the core layer. This shift from a negative to a positive PR renders the panel more susceptible to local resonance, consequently driving notable alterations in structural vibration characteristics. By carefully adjusting parameters such as the ligament–rib angle, facesheet layup mode, thickness ratio, and width–height ratio, designers can customize the CSP-TCH to mitigate resonance issues and improve stability.

## 6. Computing Efficiency

Table 9 compares the efficiency of the 3D-FEA and 2D-ERM in dynamic analysis. Notably, in free vibration and random vibration analysis, the computational time required for the 2D-ERM represents only 0.64% and 0.161% of that needed for the 3D-FEA. This highlights that the 2D-ERM, leveraging variational asymptotic homogenization, can significantly reduce calculation time in the dynamic analysis of CSP-TCH. By enhancing efficiency in analysis, the 2D-ERM offers more effective tools and methodologies for engineering design and dynamic analysis, thereby improving overall efficiency and productivity in the field.

## 7. Conclusions

This work introduces a 2D equivalent Reissner–Mindlin model (2D-ERM) utilizing VAM to examine the random vibration characteristics of CSP-TCH. Through comparison with a 3D finite element analysis, the effectiveness of the 2D-ERM in predicting vibration characteristics is confirmed. The primary research outcomes are as follows:

(1) In the free vibration analysis, the order of the first eight natural frequencies under different boundary conditions is CCCC, SSCC, FFCC, and FFCF. This ranking is in accordance with the principle that panels exhibiting higher support stiffness tend to possess higher natural frequencies. This observation is corroborated by the mode shape analysis performed on both the 2D and 3D models. Comparing the natural frequencies obtained from the 2D and 3D models, it is noted that the natural frequencies calculated by the 3D model are slightly lower than those from the 2D model. However, the overall discrepancy falls within the acceptable engineering margin of less than 7%. This suggests that the VAM-based equivalent model offers commendable accuracy and dependability in free vibration analysis, showcasing its effectiveness in capturing dynamic behavior while maintaining error levels within acceptable engineering thresholds.

(2) In the random vibration analysis, the PSD curves demonstrate a strong alignment across all four boundary conditions, with the most consistent fitting observed under FFCF boundary conditions, where the maximum displacement PSD peak error is merely 2.42%. The largest discrepancy of 4.29% appears in the CCCC boundary conditions, meeting the required engineering accuracy standards. Moreover, the peak value of displacement PSD reaches 3.73 × 10^−5^ under CCCC boundary conditions. This emphasizes that stricter boundary constraints result in decreased displacement responses but can also lead to heightened displacement PSD peak errors. The RMS curves under all boundary conditions show excellent agreement, with the RMS clouds from the 3D-FEA and 2D-ERM exhibiting similar trends and minimal discrepancies of 2.45%, affirming the efficacy of the equivalent model in accurately capturing random vibration responses.

(3) Among the considered key parameters, the rib-to-ligament thickness ratio and the width–height ratio have minimal effects on the vibration characteristics. Changes in the thickness ratio and width–height ratio primarily relate to alterations in the equivalent density. On the other hand, the impact of the ligament–rib angle on vibration characteristics predominantly arises from substantial shifts in the Poisson’s ratio of the core layer. This shift increases the likelihood of local resonance within the panel, leading to significant alterations in structural vibration characteristics. Hence, when designing and optimizing the CSP-TCH, it is crucial to carefully assess the effects of these parameter adjustments on vibration characteristics to ensure stable vibration performance. 

## Figures and Tables

**Table 1:** Material properties of CFRP facesheet.

Properties	ρ	$E_{11}$ = *E*_22_	$E_{33}$	$G_{12}$	$G_{13}$ = *G*_23_	$\nu_{12}$	$\nu_{13}$ = $\nu_{23}$
Values	1.49 g/cm^3^	41.425 GPa	14.381 GPa	15.541 GPa	3.204 GPa	0.333	0.325

**Table 2:** Comparison of first eight frequencies and free vibration modes of CSP-TCH under CCCC boundary conditions obtained from 3D-FEA and 2D-ERM.

Model	1	2	3	4
3D-FEA	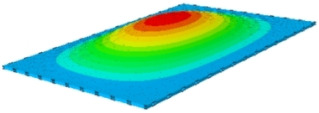	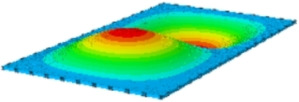	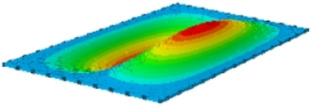	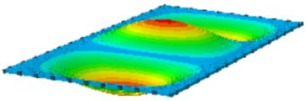
	354.17 Hz	550.19 Hz	857.76 Hz	879.66 Hz
2D-ERM	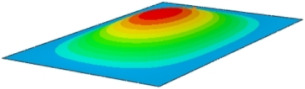	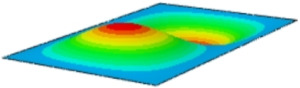	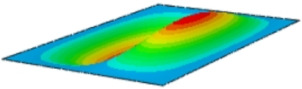	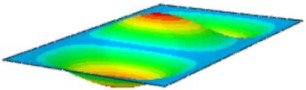
	355.3 Hz	551.99 Hz	864.01 Hz	883.05 Hz
Error	4.53%	4.39%	5.72%	4.63%
Model	5	6	7	8
3D-FEA	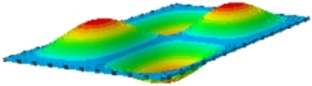	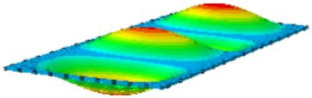	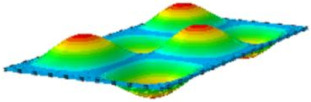	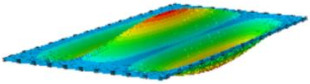
	1331.4 Hz	1341.3 Hz	1607.8 Hz	1770.2 Hz
2D-ERM	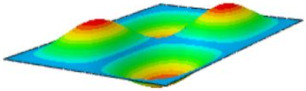	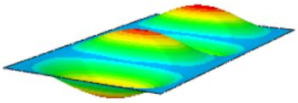	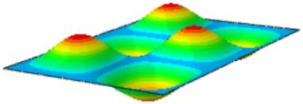	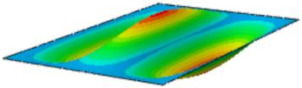
	1337.9 Hz	1350.6 Hz	1627.5 Hz	1783.1 Hz
Error	5.20%	5.45%	5.52%	5.67%

**Table 3:** Comparison of first eight frequencies of CSP-TCH under other three BCs, as predicted by 3D-FEA and 2D-ERM (unit: Hz).

Order	SSCC			FFCC			FFCF		
	3D-FEA	2D-ERM	Error	3D-FEA	2D-ERM	Error	3D-FEA	2D-ERM	Error
1	307.69	319.2	3.74%	174.6	180.44	3.34%	27.341	28.173	3.04%
2	625.05	649.21	3.87%	239.61	248.68	3.79%	90.046	93.582	3.93%
3	799.28	835.77	4.57%	480.02	498.17	3.78%	169.41	174.79	3.18%
4	1093.3	1140.8	4.34%	550.87	574.11	4.22%	304.5	316.49	3.94%
5	1093.9	1141.7	4.37%	575.86	598.89	4.00%	417.53	433.94	3.93%
6	1553.4	1623.8	4.53%	912.84	952.85	4.38%	485.78	503.76	3.70%
7	1644	1736.8	5.64%	938.66	979.65	4.37%	622.97	647.7	3.97%
8	1707.6	1793.2	5.01%	1046.3	1092.8	4.44%	659.57	687.86	4.29%

**Table 4:** Random excitation.

Frequency Range	Power Spectral Density	Acceleration Root Mean Square Value
10∼200 Hz	+6 dB/oct	14.4 g
200∼1500 Hz	1.2 g^2^/Hz	
1500∼2000 Hz	−12 dB/oct	

**Table 5:** Comparison of PSD peak errors under different cases.

BC	PSD Peak	2D-ERM	3D-FEA	Error
CCCC	U3 $m^{2}/\mathrm{Hz}$	3.57 × 10^−5^	3.73 × 10^−5^	**4.29%**
	V3 ${\left(m/s\right)}^{2}/\mathrm{Hz}$	331.09	345.66	4.22%
	A3 $g^{2}/\mathrm{Hz}$	3.08 × 10^+9^	3.21 × 10^+9^	4.05%
SSCC	U3 $m^{2}/\mathrm{Hz}$	1.32 × 10^−4^	1.27 × 10^−4^	3.8%
	V3 ${\left(m/s\right)}^{2}/\mathrm{Hz}$	631.34	654.68	3.57%
	A3 $g^{2}/\mathrm{Hz}$	3.15 × 10^+9^	3.62 × 10^+9^	3.68%
FFCC	U3 $m^{2}/\mathrm{Hz}$	9.78 × 10^−4^	10.09 × 10^−4^	3.07%
	V3 ${\left(m/s\right)}^{2}/\mathrm{Hz}$	1.25 × 10^+3^	1.27 × 10^+3^	1.57%
	A3 $g^{2}/\mathrm{Hz}$	1.49 × 10^+9^	1.51 × 10^+9^	1.32%
FFCF	U3 $m^{2}/\mathrm{Hz}$	2.82 × 10^−2^	2.89 × 10^−2^	2.42%
	V3 ${\left(m/s\right)}^{2}/\mathrm{Hz}$	825.45	831.34	0.71%
	A3 $g^{2}/\mathrm{Hz}$	2.29 × 10^+7^	2.37 × 10^+7^	3.38%

**Table 6:** Comparison of effective mass fractions of 3D-FEA and 2D-ERM under four BCs.

BC	Model	Order							
		**1**	**2**	**3**	**4**	**5**	**6**	**7**	**8**
CCCC	3D-FEA	49.22%	0	0	8.42%	0	0	0	9.67%
	2D-ERM	49.13%	0	0	8.39%	0	0	0	9.61%
	δ (%)	0.18	0	0	0.36	0	0	0	0.62
CCSS	3D-FEA	57.15%	0	0	0	9.80%	0	6.65%	0
	2D-ERM	56.59%	0	0	0	9.75%	0	6.57%	0
	δ (%)	0.28	0	0	0	0.51	0	1.2	0
FFCC	3D-FEA	68.83%	0	0	0.09%	0	0	13.22%	0
	2D-ERM	67.81%	0	0	0.08%	0	0	12.79%	0
	δ (%)	1.48	0	0	0.08	0	0	3.25	0
FFCF	3D-FEA	61.01%	0	18.85%	0	0.98%	5.59%	0	0.15%
	2D-ERM	60.15%	0	18.78%	0	0.97%	5.56%	0	0.15%
	δ (%)	1.41	0	0.37	0	1.02	0.54	0	0

**Table 7:** Comparison of the RMS cloud of CSP-TCH under different BCs predicted by 3D-FEA and 2D-ERM.

BC		U3	V3	A3
CCCC	3D-FEA	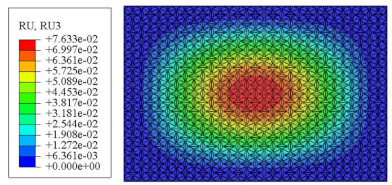	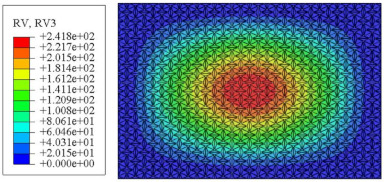	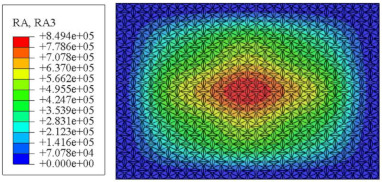
	2D-ERM	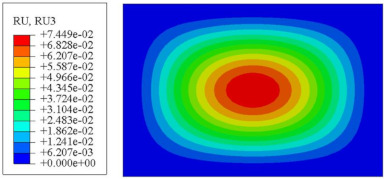	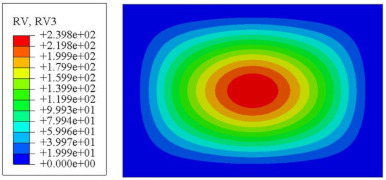	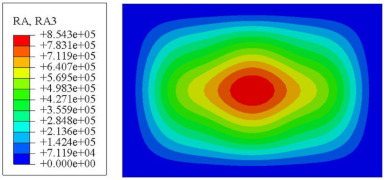
	Error	2.14%	0.83%	0.58%
SSCC	3D-FEA	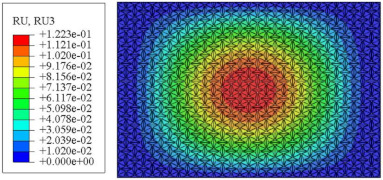	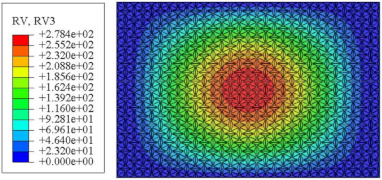	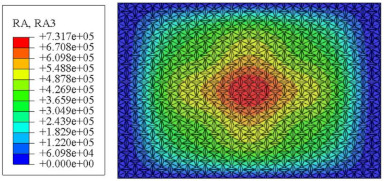
	2D-ERM	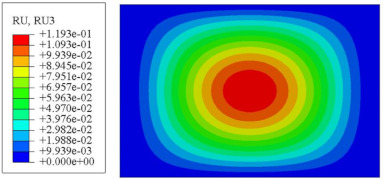	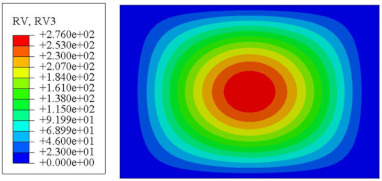	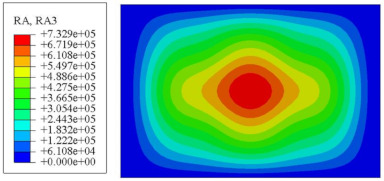
	Error	2.45%	0.86%	0.16%
FFCC	3D-FEA	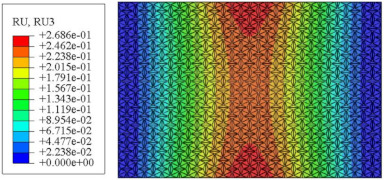	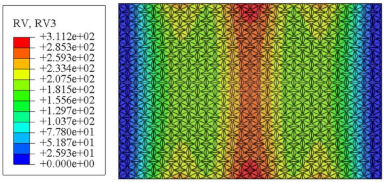	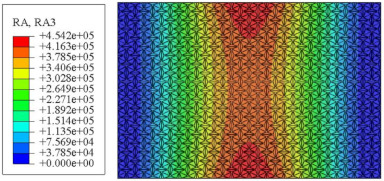
	2D-ERM	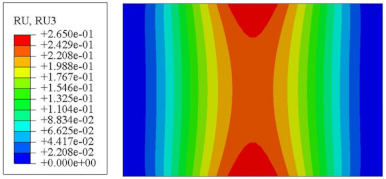	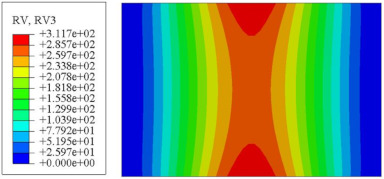	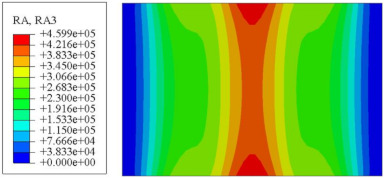
	Error	1.34%	0.16%	1.25%
FFCF	3D-FEA	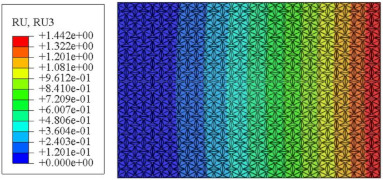	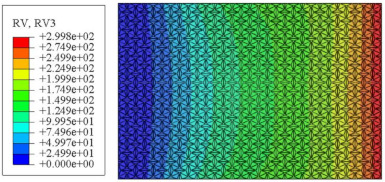	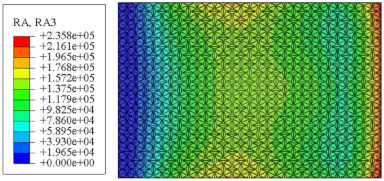
	2D-ERM	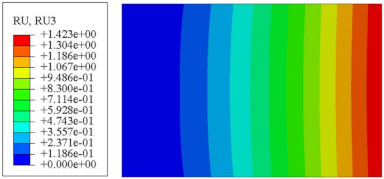	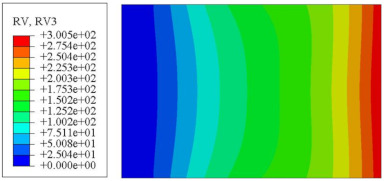	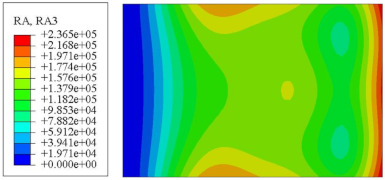
	Error	1.34%	0.23%	0.29%

**Table 8:** Comparison of change rates of vibration characteristics under the impact of individual parameters.

Parameter	Range of Variation	$|\Delta \rho^{*}|$	$\left|\Delta \nu_{1}\right|$	$\left|\Delta \nu_{2}\right|$	|Δω|	$\left|\Delta RU_{3}\right|$	$\left|\Delta U_{3}\right|$
		**(%)**	**(%)**	**(%)**	**(%)**	**(%)**	**(%)**
α	15°∼45°	9.28	**176.66**	6.33	3.30	28.86	61.33
$T_{1}/T_{3}$	0.6∼1.8	**25.72**	16.82	7.69	7.61	10.26	25.47
$L_{x}/h_{c}$	6.0∼9.0	22.32	25.88	9.58	6.80	9.49	23.19
Layup modes	ID1∼ID7	0	0	**83.11**	**43.77**	**41.12**	**76.52**

**Table 9:** Comparison of the efficiency of 3D-FEA and 2D-ERM in dynamic analysis of CSP-TCH.

Item	3D-FEA	2D-ERM	
		**3D Unit Cell**	**Equivalent Model**
Element type	C3D10	C3D10	S4R
Number of elements	371,562	12,844	3148
Free vibration analysis	1802 s	/	23 s
Random vibration analysis	4836 s	/	58 s

## Data Availability

Data available on request due to restrictions, e.g., privacy or ethical. The data presented in this study are available on request from the corresponding author. The data are not publicly available due to subsequent analyzes and publications.
